# Perceptions of Teaching Methods for Preclinical Oral Surgery: A Comparison with Learning Styles

**DOI:** 10.2174/1874210601711010109

**Published:** 2017-02-28

**Authors:** Esam Omar

**Affiliations:** Maxillofacial Surgery, Department of Oral and Maxillofacial Surgery, College of Dentistry, Taibah University, Madinah, Saudi Arabia

**Keywords:** Educational methodology of oral surgery, Preclinical course, Learning style, Teaching methods

## Abstract

**Purpose:**

Dental extraction is a routine part of clinical dental practice. For this reason, understanding the way how students’ extraction knowledge and skills development are important.

**Problem Statement and Objectives:**

To date, there is no accredited statement about the most effective method for the teaching of exodontia to dental students. Students have different abilities and preferences regarding how they learn and process information. This is defined as learning style. In this study, the effectiveness of active learning in the teaching of preclinical oral surgery was examined. The personality type of the groups involved in this study was determined, and the possible effect of personality type on learning style was investigated.

**Method:**

This study was undertaken over five years from 2011 to 2015. The sample consisted of 115 students and eight staff members. Questionnaires were submitted by 68 students and all eight staff members involved. Three measures were used in the study: The Index of Learning Styles (Felder and Soloman, 1991), the Myers-Briggs Type Indicator (MBTI), and the styles of learning typology (Grasha and Hruska-Riechmann).

**Results and Discussion:**

Findings indicated that demonstration and minimal clinical exposure give students personal validation. Frequent feedback on their work is strongly indicated to build the cognitive, psychomotor, and interpersonal skills needed from preclinical oral surgery courses.

**Conclusion:**

Small group cooperative active learning in the form of demonstration and minimal clinical exposure that gives frequent feedback and students’ personal validation on their work is strongly indicated to build the skills needed for preclinical oral surgery courses.

## INTRODUCTION

The main aim of dentistry is the retention of teeth, but in many cases extraction is an unavoidable procedure in dental care. This study focused on dental extraction because it is an essential routine procedure in dentistry. It is well documented in the literature that there are inequalities in the number of missing teeth in adults from different socioeconomic backgrounds [1, 2]. The main reasons for extractions are caries (59% of cases), periodontal disease (29.1%), orthodontic reasons (5.5%), wisdom teeth (4.6%), patient request (2.4%), trauma (1.2%), pre-prosthetic (1%), and for other reasons (6.2%) [3]. Caries and associated sequelae remain the most important cause of tooth loss throughout adult life [4].

This study aimed to determine the most effective method of teaching pre-clinical oral surgery for dental students. Dental extraction constitutes about one-third of a dental practice [1, 2] and is one of the essential procedures to learn at an undergraduate level. To determine the learning style of an individual, different learning style instruments were used. In the educational psychology literature, the Learning styles have been extensively discussed [5-10]. Students have different attitudes, and levels of motivation, about learning and teaching. There are different responses to environments of specific classroom and instruction. The more thoroughly instructors understand these differences, the better chance they have in meeting the diverse learning needs of their students [9].

Learning style is an idea obtained from psychology, and it refers to the way individuals prefer to process new information and the strategies they adopt for effective learning [7, 9, 10]. Learning style may be defined as characteristic cognitive, affective, and psychological behaviors that serve as relatively stable indicators of how learners perceive, interact with, and respond to the learning environment. There are different types of learning style models that have been presented in the literature. These include the VARK model, the ILS model, Kolb learning style, Gregorc Style Delineator and the Dunn and Dunn learning styles [5-11].

Felder and Silverman’s (1988) Index of Learning Styles (ILS) originated in the engineering sciences, are defined as the characteristic preferences and strengths in the ways, individuals take in and process information^3^. It asserts that people have inclinations (preference) along five bipolar continua: Active-Reflective, Sensing-Intuitive, Verbal-Visual, Sequential-Global, and Intuitive-Deductive. ILS provide n scores showing the strength of an individual’s preference for the indicated continuum. Individual students have relative preferences along each of the four but can learn to function in the other direction [5, 8, 11]. Students and faculty can self-score, self-interpret and self-administer this inventory. Active learners prefer practicing and performing activities in groups. Reflective learners favor working on their own, and time to think about the task before doing it is important for them. Sensing learners prefer to deal with facts, data, and experimentation with an emphasis on details. Intuiting learners thrive on working with ideas and theories, particularly new ideas and innovation. Verbal learners like to hear and discuss information, using their own words. Visual learners like to see pictures, symbols, flow charts, diagrams, and read books. Sequential learners prefer linear reasoning, systematic strategies (procedures), and material that comes to them in a constant flow (steady stream). Global learners are solid integrators and synthesizers, making intuitive discoveries and associations with seeing the general system or pattern [7-10].

The Index of Learning Styles instrument was used to assess preferences on four dimensions: active/reflective, sensing/intuitive, visual/verbal, and sequential/global [9]. The Myers-Briggs Type Indicator (MBTI)™ was also used as it is a well-known and reliable method for assessing student personality types. Created by Isabel Briggs Myers and Katherine Cooks Briggs, the inventory is based on Carl Jung’s concept of archetypes [12, 13] – NERIS Analytics Limited web-site (Free Personality Test) and The free online questionnaire of the London Business School -Defiance College). Four dimensions are used to identify an individual’s personality profile along orientation to life: (Extraverted/Introverted); perception (Sensing/Intuitive); decision making (Thinking/Feeling); and attitude to the outside world (Judgement/Perception).

The MBTI has been widely used to classify student learning styles in various disciplines [12-14]. The first two dimensions (Orientation and Perception) appear to have implications for learning [13, 14]. The MBTI provides data based on four sets of preferences [7]. These result in 16 learning styles or types. Each type is the combination of the four preferences as follows: Extraverted, Sensing, Thinking, Judger (ESTJ). For this study, students were requested to use the free online personality test [13] to correlate their personality type to their learning style. The learning styles typology, developed by Anthony Grasha and Sheryl Hruska-Riechmann was also used to determine the most common clusters of learning style [13, 14]. This typology is distinct from other educational models in that it is not based on students’ general assessment of cognitive traits or personality rather than interaction to actual classroom activities. Grasha [6] argues that this situation-specific approach is more likely to be reliable and valid. A personality type approach requires the analyst to extrapolate the outcomes to classroom settings; though, the Grasha-Riechmann typology is intended to help personnel recognize teaching procedures that location-specific learning styles [6].

### Statement of the Problem

There is a strong relationship between teaching method and preferred learning style for the achievement of effective learning [15, 16]. The harmony between the teaching method used by a lecturer and the learning styles of students is the key factor needed for effective learning. There is no accredited statement about the most effective method for teaching pre-clinical oral surgery. This study is an endeavor to set up the most effective teaching method for pre-clinical oral surgery with the students and staff at the College of Dentistry at Taibah University, Saudi Arabia. The students differ in their specific learning styles and personality and because of that, this study was conducted over five years to allow for a wide spectrum of students over five different cohorts, to establish the most effective methods of teaching pre-clinical oral surgery.

This study aimed to answer the following question: What is the most effective method for teaching pre-clinical oral surgery to develop the skills required for tooth extraction?

## METHODS

This study has been approved by Research and Ethics Committee at Taibah University with application No. TUCODREC/20151031/OMAR. It was conducted from 2011 to 2015 and involved successive cohorts of dental students in their second year. The students were taking the pre-clinical course of oral surgery (anesthesia and exodontia). The study was designed to investigate students’ preferred learning styles and to establish the most effective method of teaching this important dental course. It was conducted in the last teaching block after students had been exposed to various subjects of the course.

The first questionnaire focused on students. Sixty-eight of the 93 students (59.13% of the group) completed their questionnaire and participated in the study. The remaining students were not included. The questionnaires were distributed and collected at the end of the academic year except 2014 and 2015 when the questionnaire was distributed early in the following academic year. The second questionnaire targeted lecturers, and 77.8% of lecturers who teaching the pre-clinical oral surgery course participated in this study.

### Questionnaires

Two main questionnaires were prepared: (1) The student questionnaire consisting of questionnaires 1, 2, 3 and Part B of Questionnaire 1 (students); and, (2) The staff questionnaire consisting of questionnaires 1, 2, 3 and Part B of Questionnaire 1 (staff). In the following section, we provide more details about the questionnaires.

#### Questionnaire 1

Felder and Silverman’s (1988) ILS instrument [5, 8] was used. The results provide an indication of an individual’s learning preference and an indication of the preference profile of a group of students. A student’s learning style profile details possible strengths and tendencies or habits that might lead to difficulty in academic settings. The profile does not reflect a student’s aptitude for a particular subject, discipline or profession. The ILS is a 44-item questionnaire with two endings to a sentence that focus on an aspect of learning. A score of 1–11 is achieved with 1 and 3 demonstrating a parity (balance) along the continuum, 5 and 7 for one end of the continuum demonstrating a moderate preference, and 9 and 11show a strong preference for one or other end [5-11] (Appendix **A**). This questionnaire was distributed to both participant groups.

##### Part B of Questionnaire 1 (Students)

This questionnaire investigated the students’ beliefs regarding the best teaching methods for each subject in the pre-clinical oral surgery course. This was undertaken at the conclusion of the course based on their experience. Different active teaching methods were included in the questionnaire. Students were asked to grade each teaching method out of 100. Background information survey was used for the collection of demographic data from participants *via* asking four questions regarding sex, age, and career plan (Table **1**).

##### Part B of Questionnaire 1 (Staff)

The questionnaire evaluated the actual teaching methods and teaching aids used by the lecturers. The following methods were evaluated: lecturing, demonstration, discussion, tutorial, seminar, peer teaching, project, clinical involvement and exposure, and use of visual media. It also investigated the lecturers’ opinions about the best teaching methods for the pre-clinical oral surgery course. A blank area was left open after each teaching method so that lecturers could detail their opinion of each one. Each subject of the course was examined separately with lecturers given the opportunity to grade each method (out of 100) based on their opinion of its effectiveness.

Part B of Questionnaire 1 concluded with the same question for both groups. All participants were asked about the importance of pre-clinical courses of exodontia, and if they believe that dental students could start clinical exodontia without the preclinical course. Where respondents indicated ‘not possible,' they were then required to select one of four possible reasons for why they had given this response. The four options were:

- Students have not been previously exposed to surgical information, which would assist them in carrying out a full clinical exodontia session.
- Meeting the clinical requirements will be a challenging task as the students would need to still orient themselves to surgical practice and /or understand the surgical information of exodontia.
- Exodontia are a type of general surgical procedure, and the trainee may struggle to practice exodontia without a full understanding of surgery and the management of medically compromised patients.
- This style of teaching may be suitable for technical trainees but not for dental trainees.

#### Questionnaire 2

This questionnaire examined the participants’ predominant personality types and the relationship with their learning styles. It was distributed to both groups. A free online questionnaire based on the MBTI and Jung’s typological approach to personality [12-14] was used to determine the personality type of the sample. Extraversion – Introversion, signifies the source and direction of a person’s energy expression. For an extravert, this is mainly in the external world, while an introvert derives energy mainly from their own inner universe. The method by which someone perceives information is represented by (Sensing – Intuition). The sensing means that information from the outside universe relies on, whereas Intuition means that a person mainly relies on information from the internal or imagined world. Thinking – Feeling represents how a person processes information. Thinking means that decisions are reached mainly through logic. Feeling means that emotions rely on. Judging – Perceiving reflects person implementations and execution the data he or she have obtained. Judging means that a person organizes life events and sticks to his plans. The inclination to improvise and explore alternative options is defined as Perceiving.

All possible permutations of preferences for the four dichotomies above yield 16 different combinations or personality types. Four-letter acronym usually is used to assign each personality type, and these appear in Appendix **B**.

#### Questionnaire 3

The manual form of the learning styles typology was distributed to both groups. The Grasha-Riechmann Student Learning Styles Scale was developed to measure student learning preferences [6]. The survey can be completed by instructors and learners to evaluate teaching methods and to compare their views. It is helpful in suggesting ways for teachers to adapt and diversify their teaching methods to meet learners’ needs. The learning styles scale consists of six primary learning styles, which are present in each learner, though to varying degrees: Avoidant, Collaborative, Competitive, Dependent, Independent, and Participant. The questionnaires were collected and analyzed as appeared in Appendix **C**.

### Data Collection and Analysis

In the design of the questionnaires, care was taken to ensure that the data collected could be presented and organized systematically so that valid and accurate conclusions could be drawn from them. The Statistical Package for Social Sciences - SPSS program was used in the statistical analysis. The following statistics were used:

T-test: The t-tests used for the assessment of whether the means of the corresponding variables in the learning styles and teaching styles are statistically different from each other.

- Statistical significance - p-value: P < 0.05 was considered statistically significant [18].
- Practical significance: It was also important in this study to calculate and report measures of practical significance, known as effect size (d-value). The measure used in this study was Cohen’s *d* [17].
- Effect size (r): This is used for helping the readers to comprehend the magnitude of differences found, while criticalness (statistical significance) looks at whether the examiners are prone to be expected to chance [18].

## RESULTS AND DISCUSSION

### Teaching Methods and Learning Styles

The data collected through Questionnaire 1 were compared with the data from Questionnaire 2. The results, shown in Table **2**, indicate that both lecturers and students favored a variety of teaching/learning styles. This finding compares with various studies that have found that students are able to use a range of learning styles effectively [6, 8-11, 15, 16].

The effect sizes in this study were very small, which means that the lecturer teaching styles preference of the in this study, were to a certain extent, in equilibrium with the preferred learning styles of their students. Exceptions were noted for the Inductive/Deductive reasoning mode and the Sensory/Intuitive Perception of the information mode, which had large effect sizes. The p-value was highly significant for the Inductive/Deductive reasoning and less significant for the Sensory/Intuitive Perception.

Multiple modes of learning styles are preferred by the students, which also included the active learning mode (86.1%). This fits with the preference of lecturers for teaching with a variety of teaching style modes. There was agreement about perception of information with sensory mode (71.9% students; 78.6% lecturers); perception of sensory information with a visual mode (73% students; 80.1% lecturers), processing of information with an active mode (67.7% students; 60.3% lecturer), and understanding of information with the global mode (65.2% students; 71.1% lecturers).

In three of the variables measured, the *d*-values were small. This indicates that there is no practical significance between the two groups and that both teaching/learning modes were preferred by students and lecturers. The only large effect size was in the inductive/deductive reasoning mode (0.887), which indicates that students and lecturers preferred the opposite modes.

### Students’ Personality Types and Learning Style

The students’ personality types and their learning styles are shown in Table **3**. In this study, 65% of the students are Extraverted, and 61% of the lecturers are Extraverted in the dimension of Orientation to life. In the dimension of Perception, 72% of the students and 78% of staff are Sensing. The students were requested to use the free personality test (NERIS Analytics Limited web-site) [12-14] to correlate their personality type to their learning style. Each student independently completed the free questionnaire and sent their result to my email. The results were collected and their roles and strategies analyzed (Table **4**).

The learning styles typology [6, 15] is distinct from other educational models in that it depends on the students’ responses to actual classroom activities rather than on a general personality assessment or cognitive traits. Grasha contends that this situation-specific methodology is probably dependable and substantial. A personality-type approach has required extrapolating the results to classroom settings; whereas, the Grasha-Riechmann typology is designed to identify teaching techniques that address particular learning styles. In this study, 48% of students are Collaborative, Participant and Independent and 20% are Participant, Dependent, and Competitive. This means that the majority of students are in Clusters 2 and 3 in Grasha’s framework (Table **5**), where each dimension has been summed, divided by 34 and multiplied by 100 to yield a percentage [6]. There is a predominant match between students and departmental staff. This finding strongly supports the involvement of students in small group active learning methods, such as demonstrations and clinical exposure. The findings indicated that about half of the students are in Cluster 3, *i.e.*, they learn well from non-evaluated clinical exposure. Non-evaluated clinical exposure does not meet the criteria of evaluation of clinical courses, but it meets the criteria of preclinical courses for evaluation and assessment. The definitions personality types and learning style are in Appendix **D**.

### Teaching Methods Preferred

The teaching methods used were compared with the methods preferred by both groups. The active teaching methods that were investigated included the following: lecturing, demonstrations, discussion, tutorials, seminars, peer teaching, projects, clinical involvement and exposure, video media (Table **6**).

Lectures were most widely used in the preclinical oral surgery course. They are not the most effective method. About 50% of participants believed that active learning in the form of demonstration with minimal clinical exposure is more effective than any other methods. The results indicate that the lecturers rely heavily on the use of the traditional lecture method. This kind of education should not be regarded an effective approach to teaching skills, encourage higher-order thinking, and improve cognitive and interpersonal skills required to be developed in the preclinical course, which aims to prepare students for the clinical courses. Lecturing tends to encourage passive learning and students’ passive processing of information. It provides less opportunity for them to process and critically appraise new information offered [18]. The passive mode of receiving information is reflected in the limited problem-solving and interpretation ability demonstrated by students. Lectures do not encourage the development of interpersonal and cognitive skills.

Part B of Questionnaire 1 showed that lecturers believe that PowerPoint presentations are user-friendly, rather than being an effective method of teaching. Two of them reported that they use PowerPoint because of university regulations that encourage its use, although there are no clear regulations stating this specifically. The traditional lecture format emphasizes certain modes of learning while neglecting others because they assume that all students at the same pace, acquire the same information presented. Most students are able to learn effectively as long as the lecturer provides a blend of different modes in his or her teaching style [18]. The emphasis of this course on passive learning does not help in the achievement of its essential objectives. Active teaching like demonstration, discussions, seminars, tutorials, case studies and peer teaching are strongly recommended to meet the objectives of such courses.

### Active Teaching Methods

Results indicate that lecturers do not make effective use of active methods of teaching. Lecturers’ use active teaching methods ranged between 0% and 20%, while the preference of both groups for active learning experiences was far higher (Table **7**). This disparity indicates that lecturers like to be actively involved in the different teaching experiences, but they do not apply it. This might be identified with the fact that lecturers become used to a certain teaching style and it is difficult for them to change it. Three of the lecturers reported that giving the students information in the form of a lecture insured that all of them received the same learning information at the same time. One lecturer noted that active learning often requires dividing the students into small groups so that more staff is needed to present the information to the students. This can be time-consuming and does not guarantee that the main objectives of the course will be met. Most of them reported a combination of both passive learnings in the form of lectures, with active learning, including demonstrations and minimal clinical approaches (which do not include clinical requirements and evaluation). Both groups emphasized the importance of active clinical involvement of the students in small groups.

In response to the question about the importance of preclinical courses, and whether participants considered that dental students could start clinical exodontia without preclinical courses, most of the sample selected the choice of ‘absolutely difficult for the students to starting practicing without a preclinical exodontia course’ (92% of the student group and 87.5% of the staff group). When asked to state a reason for this response, 43.8% of all participants selected the following choice: ‘Students have not been previously exposed to surgical information, which would assist them in carrying out a full clinical exodontia session’; 38.3% selected ‘Exodontia are a type of general surgical procedure, and the trainee may struggle to practice exodontia without full understanding of surgery and the management of medically compromised patients’; 17.9% selected ‘This style of teaching may be suitable for technical trainees but not for dental trainees.’

### Recommendations

This project indicated that the most effective teaching method for this course is a demonstration and clinical exposure. For more effective teaching/learning of preclinical oral surgery, the course cannot be solely taught by PowerPoint presentation (passive teaching). It is essential to include active learning in the form of demonstrations and minimal clinical involvement with no clinical requirements. Clinical exposure should be directed towards learning rather than evaluation. Dividing students into small groups for active learning, together with demonstration and non-evaluated clinical exposure is strongly indicated. The preclinical oral surgery course should contain passive and active learning styles to achieve the objectives of such preclinical courses, *i.e.*, building the cognitive, psychomotor, professional responsibility and interpersonal skills.

The general aim of training dentists is to develop practitioners into critical thinkers, problem-solvers, lifelong learners, with skills in peer and self-evaluation and to help them acquire relevant skills that support professional development. It is clear from this study that the cooperative and active teaching/learning methods are the best way to achieve this goal.

The course should be reviewed and adapted every year so that the most suitable teaching methods can be used. Students should complete a standardized learning style questionnaire at the beginning of each year. This project indicated the importance of active learning for preclinical oral surgery, which should be considered as an essential method of teaching through the use of small groups with personal validation and frequent feedback.

## CONCLUSION

Small group cooperative active learning in the form of demonstration and minimal clinical exposure that gives a frequent feedback and students personal validation on their work is strongly indicated to build the skills needed for preclinical oral surgery courses.

**Appendix A:** Summary of Felder and Silverman’s (1988) ILS instrument.

Scale	Items	Factors
Sensing-	38,6,18,14,2	inclination of solid data (fact, data, the real)
Intuitive	10,34,26,22 42,30	word or deliberation (interpretations, theory, model)
Visual- Verbal	7,31,23,11,15 27,19,3,35,43,39	data design wanted to import data design wanted to recollections and review
Sequential- Global	20,36,44,8,12 32,24	linear / sequential or random / holistic thinking
Active- Reflective	25,1,29,5,17 37,13,9 21,33,41	activity first or reflection-first active or saved ideal or unfavorable state of mind toward gathering work

**Appendix B:** All possible permutations of preferences for the four dichotomies above yield 16 different combinations.

*The 16 personality types*			
ESTJ	ISTJ	ENTJ	INTJ
ESTP	ISTP	ENTP	INTP
ESFJ	ISFJ	ENFJ	INFJ
ESFP	ISFP	ENFP	INFP

Notes: E extraversion; I introversion; S sensing; N intuition; T thinking; F feeling.

**Appendix C:** The learning styles scale.The numbers below represent the items in the questionnaire that correspond to each of the learning style dimensions. Each column total was summed and divided by 10 to obtain the main score of each scale:

Independent	Avoidant	Collaborative	Dependent	Competitive	Participant
1……	2……	3……	4……	5……	6……
7……	8……	9……	10…..	11…..	12…..
13…..	14…..	15…..	16…..	17…..	18…..
19…..	20…..	21…..	22…..	23…..	24…..
25…..	26…..	27…..	28…..	29…..	30…..
31…..	32…..	33…..	34…..	35…..	36…..
37…..	38…..	39…..	40…..	41…..	42…..
43…..	44…..	45…..	46…..	47…..	48…..
49…..	50…..	51…..	52…..	53…..	54…..
55…..	56…..	57…..	58…..	59…..	60…..

*Competitive:* Studenrs who learn material In order to perform better than others in the course of study. They sense they must compete with other students in a class for the rewards that are provided. Preferences: Become a group leader in the discussions, Teacher centered instructional procedures, Singled out in class for doing a full job, Like to dominate discussions, Class activities where they can perform better than others. *Collaborative:* Typical of students who feel they can read by sharing thoughts and talents. They cooperate with teacher and peers and like to play with others. Preferences: Lectures with class discussions in small groups, Small seminars, Student-planned aspects of courses, Group rather than individual tasks *Avoidant:* Not enthusiastic about learning content and going to class, Do not participate with students and instructors in the classroom. They are uninterested and overwhelmed by what goes on in class. Preferences: Generally turned off by most classroom activities, Would prefer no tests, Blanket grades where everyone receives a passing grade, Does not like enthusiastic teachers. *Participant:* Good citizens in class. They enjoy going to class and take responsibility for getting the most out of a course. Want to convey part in as much of the course activity as potential. Preferences: Lectures with discussion, Opportunities to discuss material, Class reading assignments, Teachers who can examine and synthesize information well. *Dependent:* Characteristic of students who show little intellectual curiosity and who learn only what is needed. They view teacher and peers as sources of structure and support and look to authority figures for specific guidelines on what to practice and how to manage it. Preferences: Outlines or notes on the board, Clear deadlines and instructions for assignments, Teacher centered classroom methods, As little ambiguity as possible in all facets of the class. *Independent:* Students who like to think for themselves. They prefer to exercise on their own, but will heed to the minds of others in the schoolroom. Determine the content they feel is important and are sure-footed in their learning abilities. Preferences: Independent study, Prefer to go alone, Self paced instruction, Assignments that give students a chance to think independently, Projects that students can design, Student-focused rather than a teacher-centered course designs. guideline; assignments that allow understudies to think autonomously; extends that understudies can plan; understudy concentrated as opposed to educator focused course outlines [3, 18].

**Appendix D:** The definitions personality types and learning style. Definitions: 1 [18].

Competitive →	Competes with other students→	Teacher-centred, class activities
Collaborative→	Shares ideas with others→	Student-led small groups
Avoidant→	Uninterested, non-participant→	Anonymous environment
Participant→	Eager to participate→	Lectures with discussion
Dependent→	Seeks authority figure→	Clear instructions, little ambiguity
Independent→	Thinks for themselves →	Independent study and projects

**Table:** Definitions: 2 [18].

**Cluster 2** *Primary Learning Styles* Participant/Dependent/Competitive	**Cluster 3** *Primary Learning Styles* Collaborative/Participant/Independent
Personal Model/Expert/Formal Authority •Role Modelling by Illustration -Sharing Thought Processes -Sharing Personal Experiences •Role Modelling by Direct Example -Demonstrating Ways of Doing •Teacher/Coaching/Guiding Students	Primary Teaching Styles Facilitator/Personal Model/Expert •Case Studies •Guided Readings •Key Statement Discussions •Laboratory Projects •Problem-Based Learning -Group Inquiry -Guided Design -Problem-Based Tutorials •Role Plays/Simulations •Roundtable Discussion

## Figures and Tables

**Table 1:** Demographic data for student participants (n = 68).

	***N***	**%**
**Gender**		
Male	68	73
Female	0	2
**Age of participants**		
18–20 yrs.	23	33.8
21–23 yrs.	43	63.2
24–26 yrs.	2	2.9
27–30 yrs.	0	0
**Plan to pursue a career in oral surgery**		
Yes	19	27.9
No	49	72.1
**Overall satisfaction with current preclinical oral surgery course**		
Very Satisfied	5	7.4
Moderately Satisfied	32	47.1
Low Satisfaction	31	45.6
No Satisfaction	0	0

**Table 2:** Comparison of teaching methods and learning styles.

Teaching/Learning Style variable	Mode	Mean Percentage of students (N = 68)	Mean Percentage of lecturers (N = 8)	Standard deviation		P value	Cohen’s *d*	Effect Size (r)
				students	lecturers			
Perception of information	Sensory	71.9	78.6	6.8	2.2	0.0082	0.739	0.347
	Intuitive	28.1	21.4					
Perception of sensory information	Visual	73	80.1	11.4	3.9	0.0878	0.869	0.228
	Verbal	7	19.9					
Reasoning	Inductive	79.1	34.6	8.6	5.2	0.0001	3.84	0.887
	Deductive	20.9	65.4					
Processing of information	Actively	67.7	60.3	7.9	3.1	0.0124	0.697	0.329
	Reflectively	32.3	39.7					
Understanding of information	Sequentially	34.8	28.9	7.2	3.3	0.0275	0.61	0.292
	Globally	65.2	71.1					

**Table 3:** Students’ personality types and learning style.

Personality types	Modes	Mean Percentage of students N = 68 (%)	Mean Percentage of lecturers N = 8 (%)
Orientation to life	Extraverted	65	61
	Introverted	35	39
Perception	Sensing	72	78
	Intuitive	28	22
Decision making	Thinking	75	39
	Feeling	25	61
Attitude to the outside world	Judgement	26	40
	Perception	74	60

**Table 4:** Students’ roles and strategies in relation to MBTI profiles.

**Analysts**	Confident Individualism	INTJ 1.2	INTP 1.3
	People Mastery	ENTJ 3	ENTP 3.8
	Constant Improvement	INTJ 2	INTP 1.5
	Social Engagement	ENTJ 4.6	ENTP 2
**Diplomats**	Confident Individualism	INFJ 1.4	INFP 1.3
	People Mastery	ENFJ 5	ENFP 2.4
	Constant Improvement	INFJ 1.3	INFP 1
	Social Engagement	ENFJ 6.8	ENFP 2.8
**Sentinels**	Confident Individualism	ISTJ 2.3	ISFJ 1
	People Mastery	ESTJ 8.1	ESFJ 3
	Constant Improvement	ISTJ 2.2	ISFJ 1
	Social Engagement	ESTJ 7.2	ESFJ 3.4
**Explorers**	Confident Individualism	ISTP 2	ISFP 1.7
	People Mastery	ESTP 9.6%	ESFP 3
	Constant Improvement	ISTP 1.7	ISFP 2.1
	Social Engagement	ESTP 7.3	ESFP 1.2

**Table 5:** Students and lecturers by learning style clusters.

Style	Mean Percentage of Students (N = 49)	Mean Percentage of Lecturers (N = 8)
Cluster1: Dependent, Participant, Competitive	14	15
Cluster2: Participant, Dependent, Competitive	20	30
Cluster3: Collaborative, Participant, Independent	48	45
Cluster4: Independent, Collaborative, Participant	18	10

**Table 6:** Teaching methods preferred by students and lecturers in relation to actual methods used.

Teaching methods	Teaching Method Preferred by Students	Teaching Method Preferred by Lecturers	Actual teaching method
Lecturing	90%	95%	100%
Questioning	65%	80%	20%
Discussion	80%	55%	20%
Demonstration	94%	90%	0%
Seminar	15%	11%	0%
Clinical involvement	85%	90%	0%
Case study	40%	15%	0%
Peer teaching	30%	10%	0%
Project	20%	10%	5%

**Table 7:** Teaching methods in relation to graded scores given by lecturers and students.

Teaching methods	Actual methods used	Grade given for teaching methods by lecturers out of 100 (mean)	SD	Grade given for teaching methods by students out of 100 (mean)	SD	P value
Lecturing	100%	49.55	15.57	34.55	18.23	0.0511
Demonstration	20%	48.64	5.52	42.73	20.66	0.3703
Discussion	20%	5.45	1.51	1.82	2.52	0.0006
Tutorial	0%	1.36	2.34	0.91	2.02	0.6309
Seminar	0%	0.45	1.51	1.36	2.34	0.2910
Peer teaching	0%	0.91	2.02	1.82	2.52	0.3622
Project	0%	0.91	2.02	2.27	2.61	0.1861
Clinical involvement	0%	31.82	2.52	29.09	11.36	0.4461
Video media	5%	11.82	4.05	14.55	9.34	0.3848
